# Research trends of intestinal barrier in ulcerative colitis: a bibliometric analysis

**DOI:** 10.3389/fmed.2025.1657620

**Published:** 2025-10-09

**Authors:** Qi Chen, Qin Chen

**Affiliations:** ^1^The Third Affiliated Hospital of Yunnan University of Chinese Medicine, Kunming, China; ^2^Department of Proctology, Kunming Municipal Hospital of Traditional Chinese Medicine, Kunming, China

**Keywords:** ulcerative colitis, intestinal barrier, bibliometric analysis, barrier integrity, target

## Abstract

**Background:**

Ulcerative colitis (UC) is intricately associated with the dysfunction of the intestinal barrier, making the restoration of barrier integrity a promising therapeutic target. Nonetheless, there is a notable deficiency in comprehensive and systematic investigations in this domain.

**Methods:**

A total of 1,554 articles were extracted from the Web of Science Core Collection (WoSCC) and Scopus. We employed analytical tools such as CiteSpace and VOSviewer to identify leading topics and create visual representations of countries/regions, institutions, authors, keyword networks, and journals pertinent to this field of study.

**Results:**

Since 2020, there has been a marked increase in the annual publication rate, with China accounting for 72.13% of the total output. In terms of international collaboration patterns, China and the United States form the core cooperative system, which has served as the foundation for establishing a globalized research collaboration network. Frequently occurring keywords underscore the application of traditional Chinese medicine, repair of tight junctions, and the NF-κB/oxidative stress pathway. Contemporary studies are increasingly emphasizing targeted delivery and clinical translation, with epithelial barriers and organoid models emerging as prominent research foci. Influential publications have primarily concentrated on the pathological mechanisms and experimental models related to UC, while the journal “Inflammatory Bowel Diseases” has proven to be an essential platform for the dissemination of knowledge within this sphere.

**Conclusion:**

The intestinal barrier has surfaced as a significant therapeutic target for UC, fostering a multidisciplinary research network. Future endeavors should aim at enhancing the linkage between mechanistic studies and clinical implementations to yield improved practical outcomes. Additionally, promoting a more equitable distribution of international collaborations is vital for the advancement of targeted intervention strategies.

## 1 Introduction

Ulcerative colitis (UC) is a long-lasting and advancing inflammatory bowel disorder marked by ongoing inflammation and ulcerative lesions, primarily targeting the colon and rectum. Clinically, individuals diagnosed with UC commonly exhibit symptoms such as diarrhea, abdominal discomfort, weight reduction, and anemia. As the condition advances, these manifestations generally intensify over time. Although the complete pathogenesis of UC remains unclear, it is significantly linked to genetic predisposition, dysregulation of the immune response, and alterations in gut microbiota composition (1, 2). Studies have demonstrated that a compromised intestinal barrier function is fundamental to the onset of UC. In particular, the disruption of tight junctions (TJs) leads to increased intestinal permeability, which can instigate uncontrolled inflammatory reactions (3, 4). Consequently, preserving the integrity of the intestinal barrier is essential for both the prevention and management of UC. Recently, efforts to restore the intestinal barrier have emerged as a central theme in the development of anti-colitis therapies (5, 6). Currently, Western medicines used for the treatment of UC, such as 5-aminosalicylic acid (5-ASA), not only demonstrate good clinical efficacy but also exhibit protective effects on intestinal barrier function. Studies have shown that 5-ASA treatment alleviates dextran sulfate sodium (DSS)-induced colitis in mice and significantly increases the protein expression levels of junctional adhesion molecule-A (JAM-A) and Occludin. Furthermore, 5-ASA promotes the proliferation of colonic epithelial cells, thereby enhancing intestinal barrier function (7). An oral 5-ASA delivery system has demonstrated significant alleviating effects in a DSS-induced colitis model, upregulating the expression of claudin-1, Occludin, and zonula occludens-1 (ZO-1) in NCM460 cells and enhancing intestinal epithelial barrier integrity (8).

In recent years, there has been a steady advancement in research pertaining to UC and the intestinal barrier, evidenced by a yearly rise in pertinent publications. Notable progress has been achieved in comprehending molecular mechanisms, inflammatory processes, and therapeutic approaches, providing significant insights beneficial for both clinical practice and foundational research. Comprehending current research status and hotspots in this field facilitates deeper exploration of UC pathogenesis and identification of potential therapeutic targets. Bibliometrics has emerged as an essential methodological approach, enabling researchers to systematically accumulate knowledge and synthesize evidence. Through quantitative analysis and visualization techniques, this approach provides novel perspectives for identifying research trends and forecasting future directions from mathematical and statistical standpoints (9).

To improve the interpretability of bibliometric data, we integrated these indicators with expertise, which enables a systematic investigation of transitions in intestinal barrier research related to UC. Co-citation analysis is employed to identify influential knowledge bases and foundational topics, while keyword co-occurrence and burst detection reveal emerging research hotspots and shifts in scientific attention. This transition has evolved from early mechanistic inquiries into tight junction integrity and inflammatory signaling to recent emphases on gut microbiota modulation, organoid modeling, and translational interventions.

**Figure 1:**
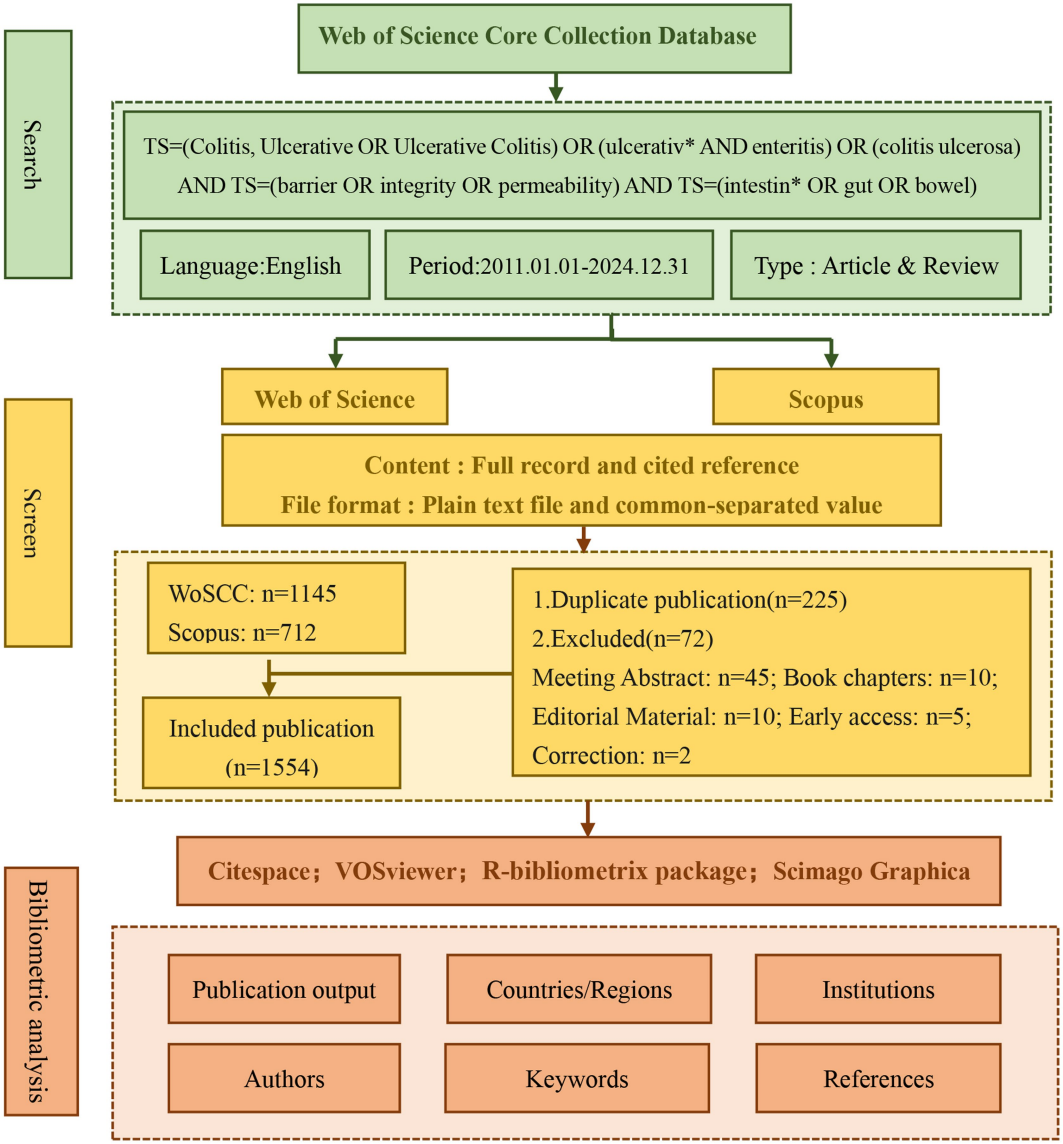
Flowchart of the literature screening process.

Therefore, this study aims to utilize bibliometric visualization tools to analyze the research status, key areas of focus, and future directions of the intestinal barrier in UC from 2011 to 2024. The findings will be presented through a knowledge map, providing insights into the evolving landscape of this field (Figure 1).

## 2 Methods

### 2.1 Data sources

In this study, the timeframe of the articles evaluated spanned from January 1, 2011, to December 31, 2024. Data were sourced from the Web of Science Core Collection (WoSCC) and Scopus. We used advanced search to identify publications relevant to UC with the following query: TS = (Colitis, Ulcerative OR Ulcerative Colitis) OR (ulcerativ* AND enteritis) OR (colitis ulcerosa) AND TS = (barrier OR integrity OR permeability) AND TS = (intestin* OR gut OR bowel) AND Language: (English). Timespan: 2011–2024. And the types of documents included were both articles and reviews. Following the selection process and the removal of duplicates, a total of 1,554 pertinent articles were ultimately identified. In the “TOPIC” search field, results were restricted to publications containing the search term in their titles, ensuring identification of scientific outputs exclusively focused on UC. Furthermore, we employed manual screening to precisely identify literature primarily focused on intestinal barrier research in UC. Our classification is principally determined by the primary outcome measures delineated in the study title, abstract, and full-text documentation. For instance, investigations that explicitly designate “ulcerative colitis” as the subject of inquiry and predominantly assess intestinal barrier-related endpoints in either UC human cohorts or murine models are categorically classified as UC intestinal barrier research.

### 2.2 Analysis methods

We acquired literature records in a plain text format for subsequent analysis. Utilizing VOSviewer version 1.6.20 and CiteSpace version 6.2. R4 software, we performed comprehensive examinations of countries, institutions, authors, keywords, and journals. Among these tools, CiteSpace, designed by Professor Chaomei Chen, is recognized as a prominent software for bibliometric analysis and visualization. It facilitates a more profound comprehension of research trends and dynamics in this domain through analyses such as institutional collaboration, keyword clustering, and timeline visualization of keyword clusters (10). In the constructed institutional and keyword co-occurrence networks, the dimensions of the nodes indicate the frequency of occurrence or citation, while the thickness of the connecting lines denotes the strength of collaboration. Furthermore, VOSviewer provides various visualization modes, encompassing co-authorship, countries/regions, and supports both network and density visualizations (11). Additionally, we employed the bibliometrix package version 4.3.2 (12) in R for frequency statistics regarding countries, encompassing collaboration frequency and intensity, and utilized Scimago Graphic version 1.0.48 (13) for visual representation. The integration of these analytical tools offers substantial support for a thorough understanding of the current state and trends in research pertaining to UC and the intestinal barrier.

## 3 Results

### 3.1 Analysis of the publications

**Figure 2:**
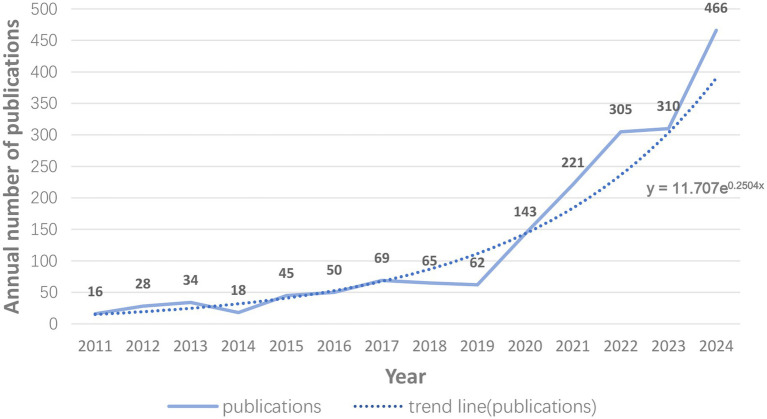
Trends in publications. Trends in the annual number of publications related to intestinal barrier and UC research.

Figure 2 depicts the yearly publication trends pertaining to intestinal barrier and UC research spanning from 2011 to 2024. A comprehensive analysis yielded 1,554 pertinent articles sourced from the WoSCC and Scopus. The line graph illustrates the increasing trend of research outputs within this domain, with the trend line highlighting a steady upward trajectory throughout the years. The function y = 11.707e^0.2504x^ exemplifies a canonical exponential growth model, as evidenced by its distinct upward trajectory.

From 2011 to 2020, the annual output of publications displayed a gradual increase, with yearly figures fluctuating between 16 and 62 articles, culminating in a total of fewer than 400 publications during this period. However, a marked increase in the number of publications has been observed since 2021, particularly in the years 2022 and 2024, signifying a heightened level of research activity and an escalating interest in this domain. Between 2021 and 2024, approximately 1,000 publications were generated, which represents 75.09% of the cumulative output over the preceding 14 years. Significantly, the annual publication count rose sharply from 143 in 2020 to 446 in 2024, exemplifying a trend of rapid growth. Collectively, the data illustrates the swift advancement in the study of intestinal barriers and UC, underscoring the increasing enthusiasm for research in this field.

### 3.2 Analysis of the contribution of major countries

In total, a cohort of 79 nations engaged in investigations pertaining to the intestinal barrier and UC from 2011 to 2024. The data elucidated in Table 1 highlights significant disparities in both the volume of scholarly publications and the academic impact among the leading 10 countries. China occupies the foremost position with 1,121 research articles, whereas the United States ranks second with 179 publications. In terms of collaborative strength, the centrality score emerges as a pivotal metric. The IRELAND exhibits the highest centrality score at 0.86, trailed closely by the FRANCE with a score of 0.72.

**Table 1:** The top 10 countries/regions in terms of publications.

Rank	Country	Count	Centrality	Year	Citations	TLS
1	China	1,121	0.22	2012	18,090	1808
2	USA	179	0.53	2011	7,330	791
3	Germany	56	0.38	2011	2028	153
4	South Korea	43	0	2011	599	120
5	Canada	37	0.22	2011	1,616	160
6	Japan	35	0.41	2011	1,234	103
7	Italy	31	0.32	2013	505	74
8	India	29	0.06	2012	429	79
9	France	26	0.72	2011	3,332	370
10	Brazil	19	0.3	2016	288	60

The research contributions originating from China undeniably represent a significant influence in the academic community, as evidenced by the remarkable total citation count of 18,090 for its publications, which substantially surpasses that of other nations. In contrast, while the United States has produced a lower volume of research papers, its citation tally of 7,330 highlights the considerable impact and relevance of its scholarly work. The total link strength (TLS) metrics outlined in the accompanying table illuminate the collaborative relationships among various countries, with both China and the United States standing out in these areas, indicating their preeminent positions within the international academic sphere. Furthermore, the associated map provides a visual representation of the intensity of academic partnerships and research outputs between nations, employing lines and dots of diverse colors to convey this information. According to the figure, the collaborative efforts between China and the United States are the most prolific, amounting to a total of 65 partnerships, which signifies a robust level of collaboration in this domain. This is followed by the collaborative interactions between the United States and Canada, which consist of 11 partnerships. Thus, this visualization underscores China’s pivotal role in the global collaborative network, as well as its strong alliances with other global regions, particularly with the United States and Canada.

**Figure 3:**
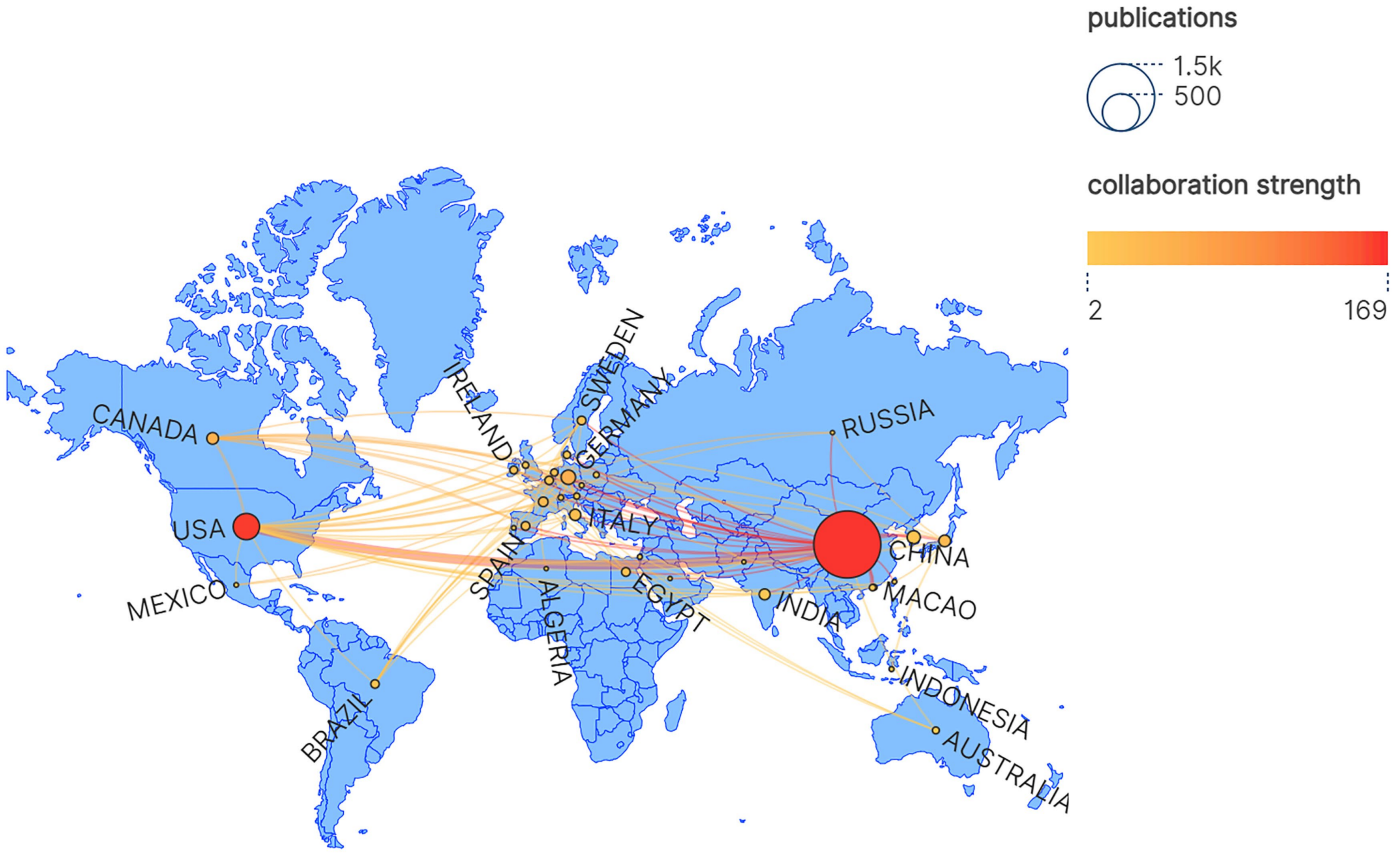
Countries’ collaboration world map. The blue areas represent different countries, with the dots within them reflecting the number of publications from each country. The larger the dot, the greater the number of publications from that country. The thickness of the lines indicates the strength of collaboration between countries, with thicker lines representing higher frequencies of cooperation.

China and the United States have both played pivotal roles in advancing the understanding of the intestinal barrier and UC, simultaneously establishing a strong international collaborative framework. Remarkably, the extent and influence of research originating from China surpass those of other nations, thereby establishing it as a prominent leader in global research partnerships (Figure 3).

### 3.3 Analysis of major institutions

**Figure 4:**
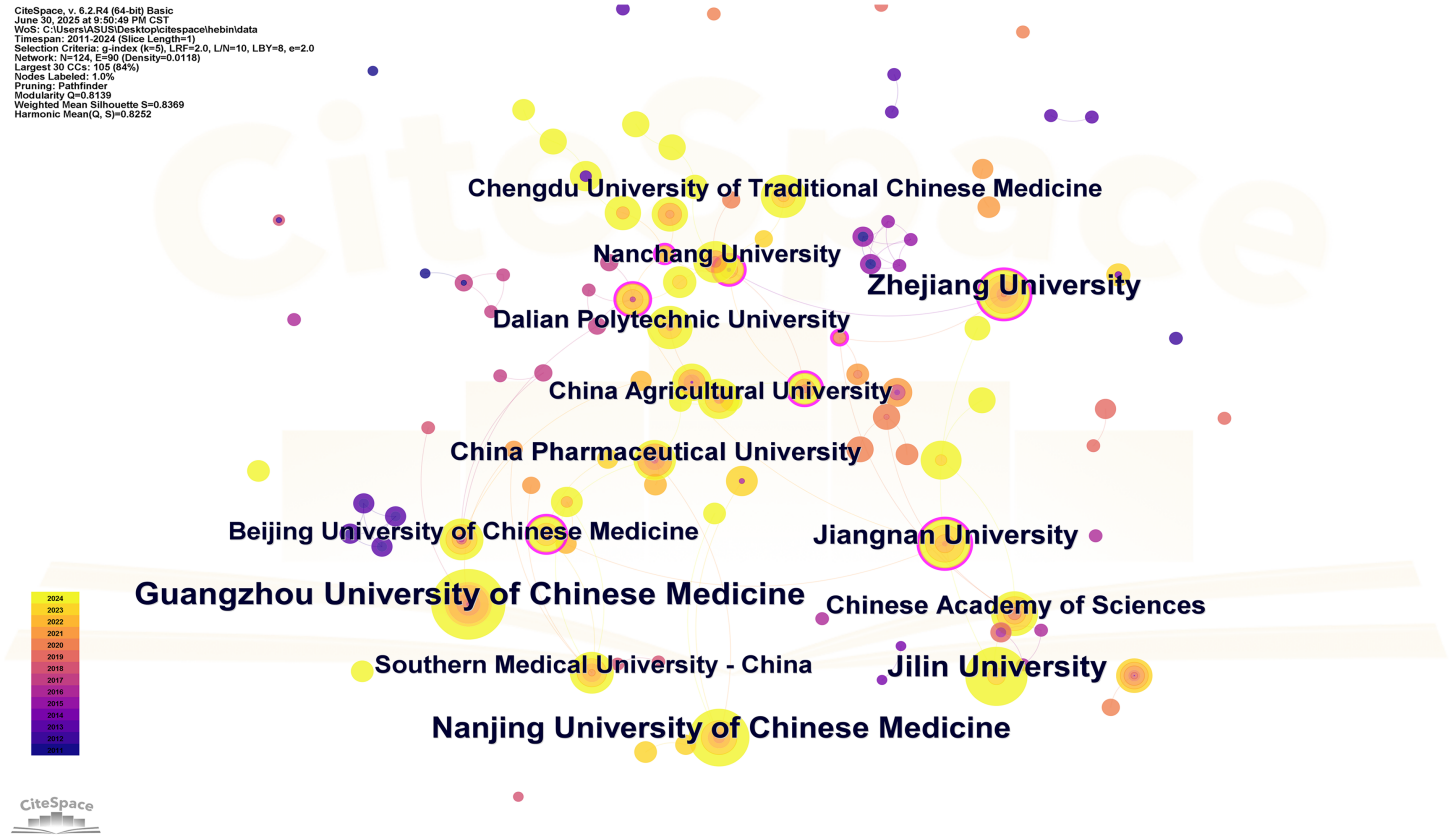
Institutional cooperation network. Each node represents a distinct research institution, with the size of the node positively correlated to its publication output—the larger the node, the more prolific the institution’s academic contributions. The arcs connecting the nodes signify collaborative relationships between institutions, where the thickness of the arcs reflects the intensity of collaboration; thicker arcs indicate higher frequencies of cooperation. Isolated nodes (those without connections) represent institutions that have not established collaborative ties with others. Nodes with purple edges denote institutions with high centrality within the network, signifying their pivotal role in the collaborative framework.

This research has pinpointed a total of 2,589 institutions globally that are actively contributing to the pertinent academic literature. The top ten institutions ranked by publication output are presented in Table 2, all of which are universities or research organizations situated in China. Notably, Guangzhou University of Chinese Medicine ranks first in terms of research output with 49 publications, although its influence within the collaborative network is relatively limited. In Table 3, research institutions from different countries with centrality greater than or equal to 0.16 are listed. University of Macau exhibits the highest centrality score of 0.28, trailed by the Chengdu University with a score of 0.25. Furthermore, several institutions recorded a considerable number of publications in 2020, marking this year as particularly productive and contributing to the ongoing academic advancement in this area (Figure 4).

**Table 2:** The top 10 institutions in terms of publications.

Rank	Institution	Publications	Centrality	Year	Citations
1	Guangzhou University of Chinese Medicine	49	0.02	2018	1,018
2	Jilin University	44	0.03	2017	420
3	Nanjing University of Chinese Medicine	39	0.03	2020	242
4	Zhejiang University	30	0.19	2015	1,295
5	Jiangnan University	28	0.05	2020	551
6	China Pharmaceutical University	25	0.1	2018	709
7	Chinese Academy of Sciences	23	0.03	2020	659
8	Huazhong University of Science & Technology	21	0.07	2015	414
9	Nanchang University	21	0.01	2020	317
10	Chengdu University of Traditional Chinese Medicine	21	0.01	2021	298

**Table 3:** Top 10 research institutions in terms of centrality.

Rank	Institution	Publications	Centrality	Year
1	University of Macau	13	0.28	2022
2	Chengdu University	5	0.25	2022
3	Cairo University	3	0.24	2022
4	Ministry of Agriculture and Rural Affairs	16	0.22	2015
5	Shanghai Jiao Tong University	13	0.2	2013
6	Zhejiang University	30	0.19	2015
7	Capital Medical University	10	0.18	2020
8	Academy of Military Medical Sciences - China	3	0.18	2017
9	Beijing University of Chinese Medicine	19	0.16	2017
10	Tianjin University of Traditional Chinese Medicine	6	0.16	2017

### 3.4 Analysis of the main authors

Author analysis plays a pivotal role in pinpointing leading scholars within a specific research domain. In Table 3, the ten most prolific authors in the area of intestinal barrier and UC research are presented, along with their primary co-cited authors, thereby underscoring their scholarly output and impact. Wei Chen from Sir Run Run Shaw Hospital, affiliated with Zhejiang University School of Medicine, occupies the foremost position with 14 publications and an impressive citation count of 290. Noteworthy is Professor Yitao Wang from the University of Macau, who has produced a comparable volume of publications (n = 9) to other prolific authors, yet boasts a remarkable citation tally of 327, indicating his considerable scholarly influence in this arena. Among the co-cited authors, the most frequently cited is Professor Siew C Ng from the University of Hong Kong, with 190 citations, closely followed by Professor Malin E V Johansson from Huazhong University of Science and Technology, who has received 187 citations.

**Figure 5:**
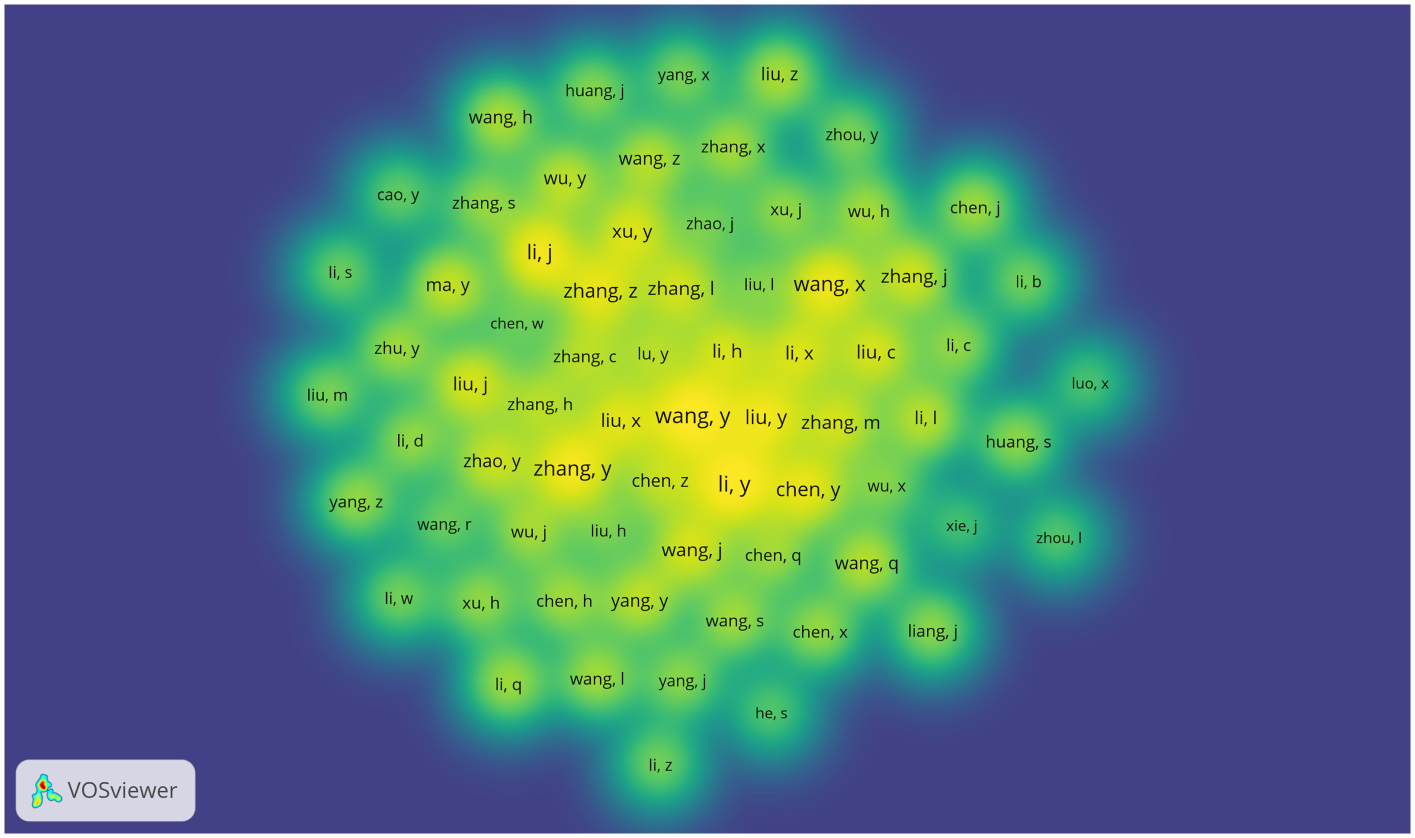
Density map of authors. The color gradient transitions from deep blue to bright yellow, symbolizing the gradient change in author density. Deep blue areas represent regions with lower author density, where the frequency of collaboration among authors is relatively low. Conversely, yellow areas indicate higher author density, reflecting a higher frequency of collaboration among these authors.

In order to provide a more intuitive understanding of the interactions and collaborations among core authors, this investigation employed VOSviewer to visualize and analyze the literature pertaining to 10,620 researchers. Utilizing Price’s Law, the criterion for defining core authors is mathematically represented as M = 0.749 (Nmax)^1/2. Here, M signifies the minimum number of publications necessary to be classified as a core author, while Nmax indicates the publication count of the most prolific researcher. By applying a threshold of over three publications, a total of 203 core authors were identified. The author density visualization presented in Figure 5 illustrates the co-occurrence relationships and distribution patterns of these key authors within the same research domain. It is noteworthy that the centrality score for each author remains below 0.1. Y Li is prominently represented within the collaborative framework, with direct associations with as many as 37 authors, suggesting that she maintains a relatively close and extensive academic collaborative network in this discipline (Table 4).

**Table 4:** The top 10 authors and co-cited authors in terms of publications.

Rank	Authors	Counts	H-index	Rank	Co-cited author	Citations	H-index
1	Li, Y	49	36	1	Ng, Sc	190	88
2	Wang, Y	56	41	2	Johansson, Mev	187	54
3	Zhang, Y	42	47	3	Neurath, Mf	186	63
4	Chen, Y	38	2	4	Ungaro, R	184	42
5	Wang, X	37	55	5	Wirtz, S	157	31
6	Li, J	35	6	6	Kaplan, Gg	129	76
7	Liu, Y	33	18	7	Kobayashi, T	116	42
8	Li, X	29	77	8	Turner, Jr	110	85
9	Wang, J	32	6	9	Danese, S	108	118
10	Zhang, J	27	22	10	Liu, Y	104	18

### 3.5 Analysis of co-cited references and references bursts

**Figure 6:**
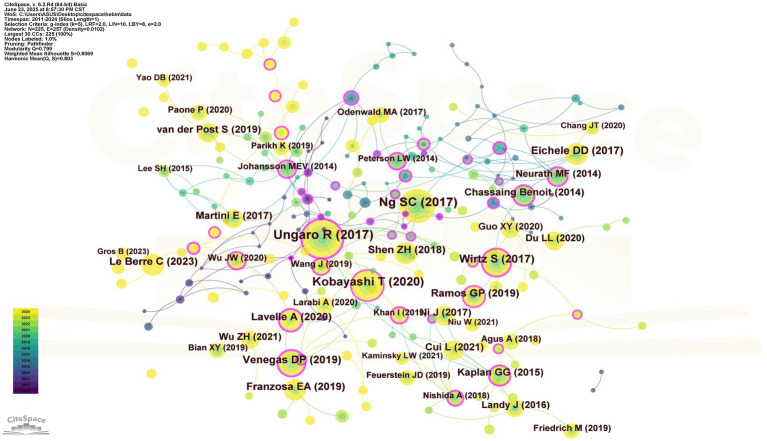
The Co-cited References network. The size of the nodes corresponds to the total citation frequency of the references, with larger nodes indicating higher citation counts. The thickness of the connecting lines represents the strength of the co-citation relationships; thicker lines denote stronger associations. The red tree rings illustrate the citation burst intensity, where greater thickness signifies more pronounced academic influence of the respective literature during specific periods.

**Figure 7:**
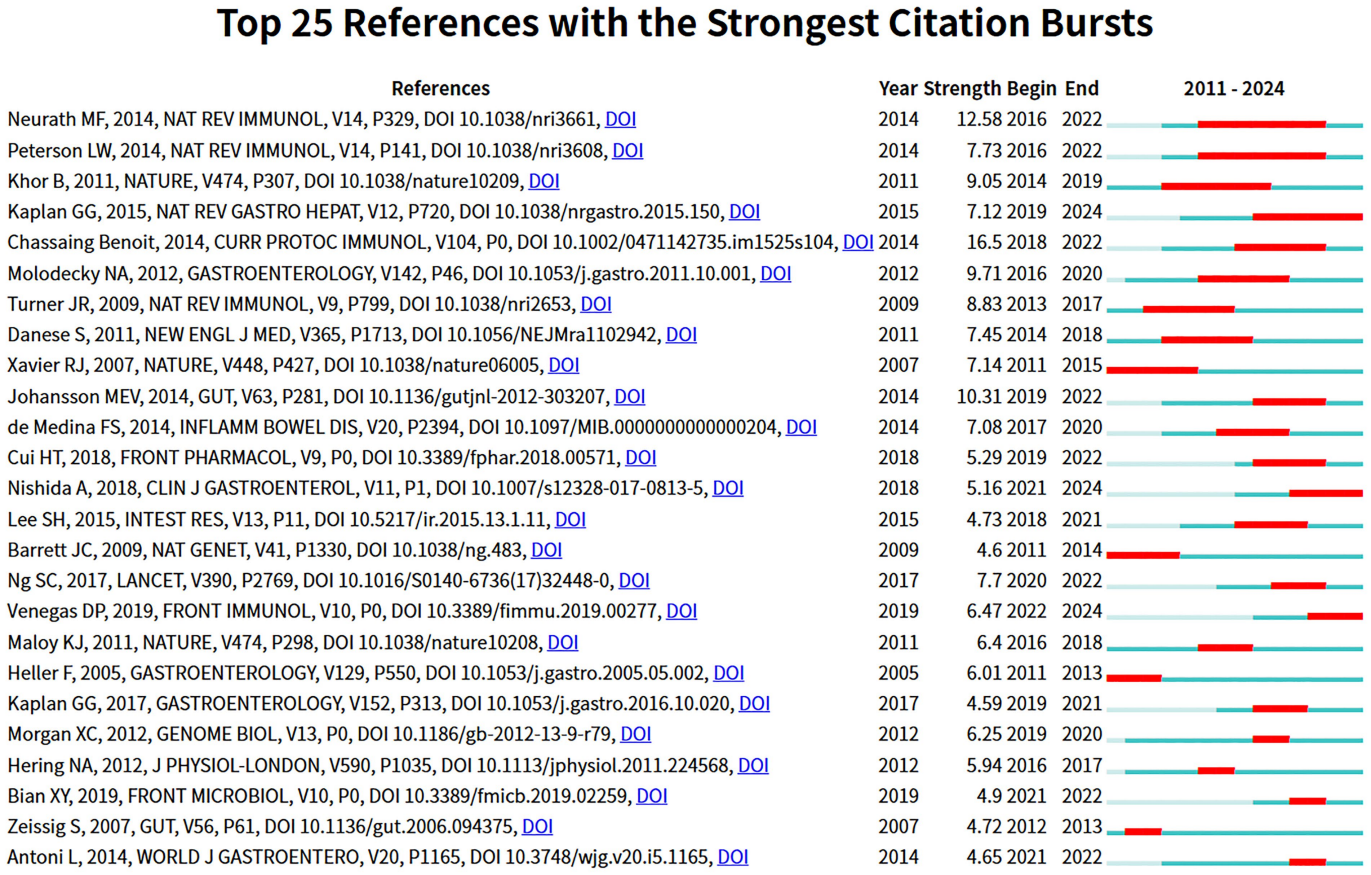
Top 25 references with the strongest citation bursts. The blue line represents the time span from 2011 to 2024, while the red line indicates the citation activity of papers within specific time intervals. A higher burst intensity signifies a rapid increase in citation counts during that particular period.

**Figure 8:**
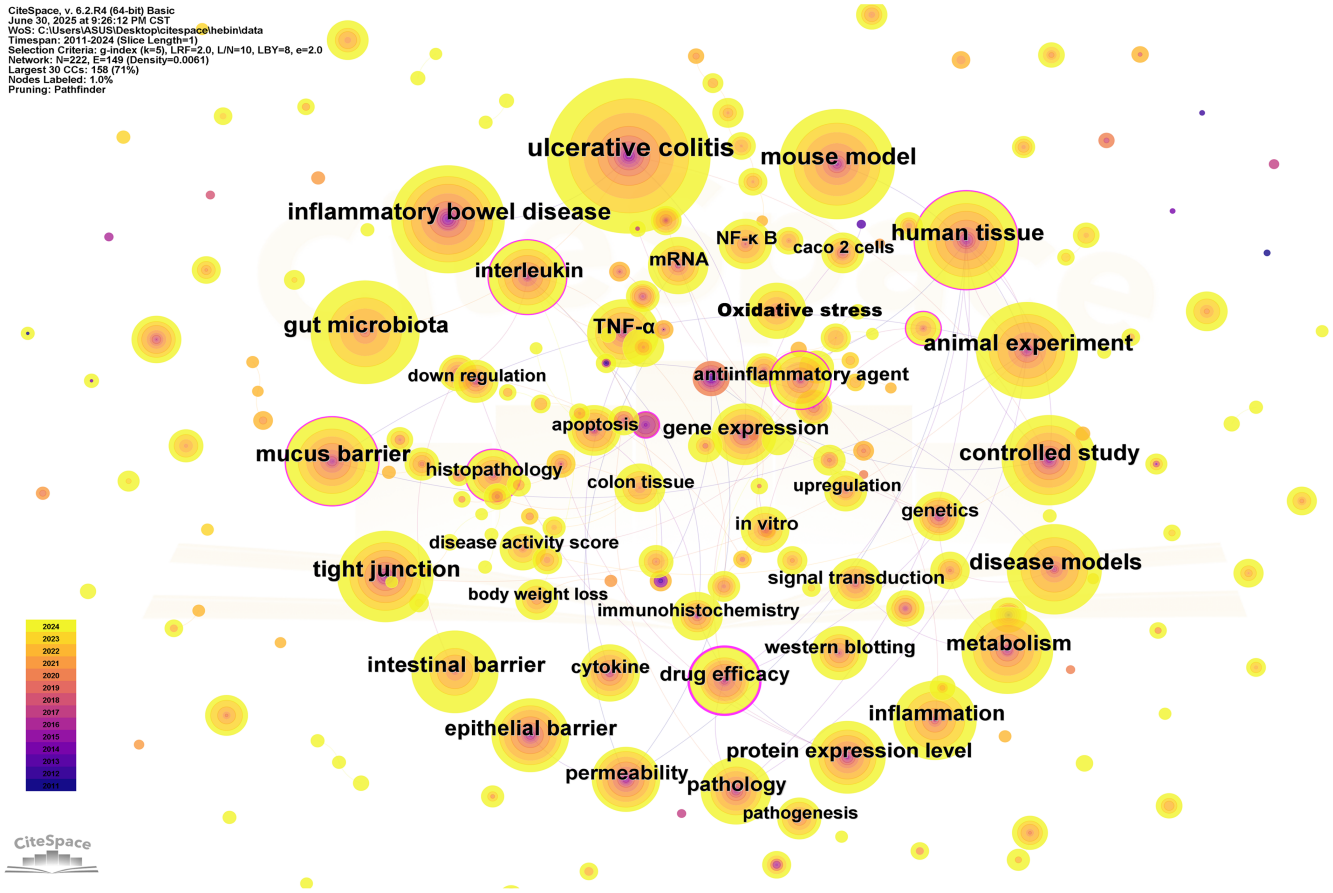
The Keywords network. The size of the nodes represents the frequency of keyword occurrences, with larger nodes indicating higher frequencies. The lines connecting the nodes denote keyword co-occurrences, where thicker lines reflect greater co-occurrence frequencies. A purple border around a node signifies high central.

A total of 240 co-cited references were identified in this study. Literature retrieval was conducted through the WoSCC, and the data were refined by excluding author self-citations. Ultimately, the top 10 most frequently cited core references were selected and presented in Table 5. The article titled “Worldwide incidence and prevalence of inflammatory bowel disease in the 21st century: a systematic review of population-based studies” authored by Ng, Siew C is notable for its 3,868 citations, underscoring its significant influence within the discipline. The impact factors and Journal Citation Reports (JCR) quartile classifications for all references were ascertained using the Chinese Academy of Sciences Journal Partition Table (2025 edition). Figure 6 illustrates a visualization network of co-cited references, where the work by Johansson, Mev attained the highest centrality score of 0.76, indicating its essential role in the intellectual framework of this research area. Furthermore, a publication exhibiting significant citation bursts typically indicates its landmark status in related inquiries, reflecting a strong acknowledgment from the academic community regarding its contributions. For example, the article “Cytokines in inflammatory bowel disease” by Markus F Neurath ranks at the forefront with a citation burst strength of 12.58, emphasizing its remarkable prominence in the year 2014. Concurrently, the research conducted by Gilaad G Kaplan entitled “The global burden of IBD: from 2015 to 2024” demonstrates a sustained citation burst, indicating its lasting and significant impact on subsequent research endeavors (Figures 7, 8).

**Table 5:** The top 10 references in terms of citations.

Rank	Co-cited references	Year	Citations	IF	JCR	Centrality
1	Ng SC, Shi HY, Hamidi N, Underwood FE, Tang W, Benchimol EI, Panaccione R, Ghosh S, Wu JCY, Chan FKL, Sung JJY, Kaplan GG. Worldwide incidence and prevalence of inflammatory bowel disease in the 21st century: a systematic review of population-based studies. Lancet. 2017,390(10114):2769–2,778.	2017	3,868	88.5	Q1	0.05
2	Parada Venegas D, De la Fuente MK, Landskron G, González MJ, Quera R, Dijkstra G, Harmsen HJM, Faber KN, Hermoso MA. Short Chain Fatty Acids (SCFAs)-Mediated Gut Epithelial and Immune Regulation and Its Relevance for Inflammatory Bowel Diseases. Front Immunol. 2019,10:277.	2019	2,444	5.9	Q2	0.48
3	Ungaro R, Mehandru S, Allen PB, Peyrin-Biroulet L, Colombel JF. Ulcerative colitis. Lancet. 2017,389(10080):1756–1770.	2017	1872	88.5	Q1	0.59
4	Franzosa EA, Sirota-Madi A, Avila-Pacheco J, Fornelos N, Haiser HJ, Reinker S, Vatanen T, Hall AB, Mallick H, McIver LJ, Sauk JS, Wilson RG, Stevens BW, Scott JM, Pierce K, Deik AA, Bullock K, Imhann F, Porter JA, Zhernakova A, Fu J, Weersma RK, Wijmenga C, Clish CB, Vlamakis H, Huttenhower C, Xavier RJ. Gut microbiome structure and metabolic activity in inflammatory bowel disease. Nat Microbiol. 2019,4(2):293–305.	2023	1,169	19.4	Q1	0.05
5	Lavelle A, Sokol H. Gut microbiota-derived metabolites as key actors in inflammatory bowel disease. Nat Rev. Gastroenterol Hepatol. 2020,17(4):223–237.	2020	1,088	51.0	Q1	0.46
6	Wirtz S, Popp V, Kindermann M, Gerlach K, Weigmann B, Fichtner-Feigl S, Neurath MF. Chemically induced mouse models of acute and chronic intestinal inflammation. Nat Protoc. 2017,12(7):1295–1,309.	2017	993	16	Q1	0.22
7	Kobayashi T, Siegmund B, Le Berre C, Wei SC, Ferrante M, Shen B, Bernstein CN, Danese S, Peyrin-Biroulet L, Hibi T. Ulcerative colitis. Nat Rev. Dis Primers. 2020,6(1):74.	2020	871	60.6	Q1	0.16
8	Eichele DD, Kharbanda KK. Dextran sodium sulfate colitis murine model: An indispensable tool for advancing our understanding of inflammatory bowel diseases pathogenesis. World J Gastroenterol. 2017,23(33):6016–6,029.	2017	693	5.4	Q2	0.09
9	Ramos GP, Papadakis KA. Mechanisms of Disease: Inflammatory Bowel Diseases. Mayo Clin Proc. 2019,94(1):155–165.	2021	615	6.7	Q1	0.1
10	Shen ZH, Zhu CX, Quan YS, Yang ZY, Wu S, Luo WW, Tan B, Wang XY. Relationship between intestinal microbiota and ulcerative colitis: Mechanisms and clinical application of probiotics and fecal microbiota transplantation. World J Gastroenterol. 2018,24(1):5–14.	2018	435	5.4	Q1	0.02

### 3.6 Analysis of keywords

Keywords serve as a succinct representation of the essential content within scientific literature delineating primary themes and providing a broad overview of the research domains. A comprehensive examination of these keywords can elucidate focal areas developmental trajectories and transformations in research interests within a specific discipline. Table 5 enumerates the top 20 most prevalent keywords in the field. Leading the list is “ulcerative colitis,” appearing 1,349 times followed by “mouse model” with 721 mentions and closely trailing is “inflammatory bowel disease,”which occurs 689 times. By scrutinizing these frequently cited keywords we can discern the dominant trends nascent research themes and evolving interests in this area of study thereby yielding significant insights that may inform future scientific endeavors.

**Figure 9:**
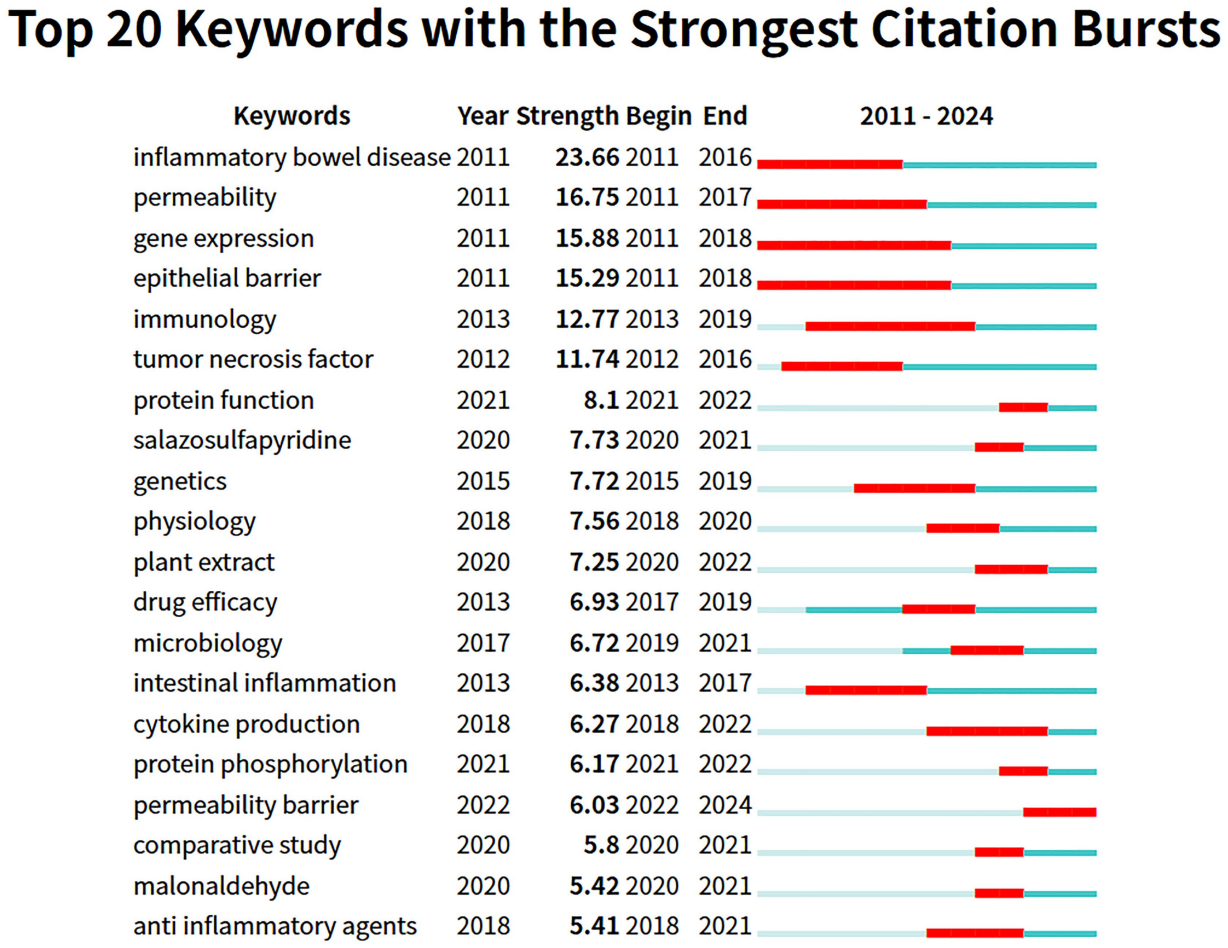
Top 20 Keywords with the strongest citation bursts. The blue line represents the period from 2011 to 2024, while the red line indicates the citation activity of papers within specific time intervals. A higher burst intensity signifies a rapid increase in citation counts during that particular period.

**Figure 10:**
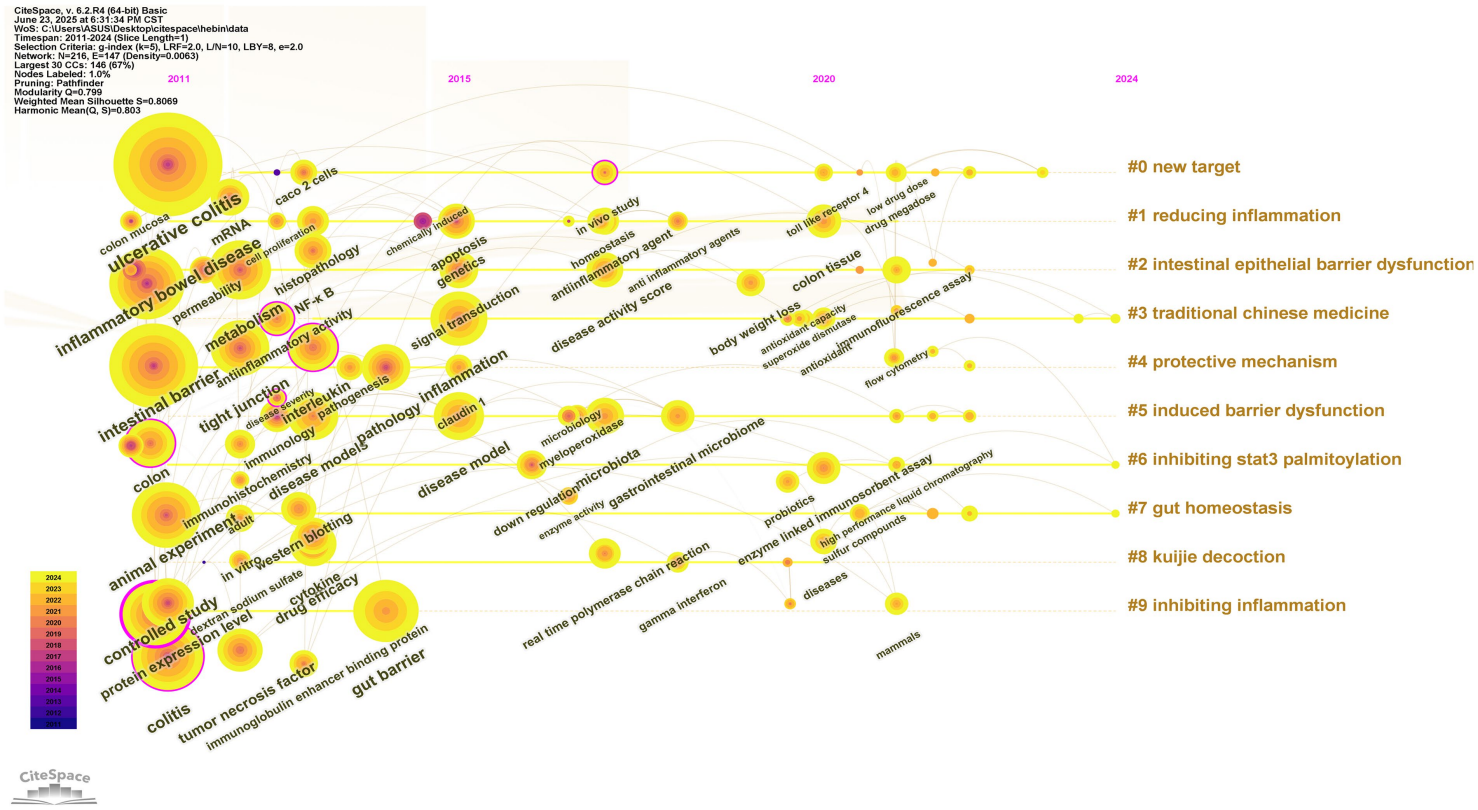
Timeline view of keywords. The left side represents the earliest nodes, while the right side corresponds to the most recent nodes. The horizontal position of each node is constrained by its respective time zone, yet vertical connections are permitted to link nodes across different time zones. These connections illustrate the co-occurrence relationships of keywords among distinct clusters.

Following the exclusion of irrelevant high-frequency terms, “gut microbiota” surfaced as the most significant keyword. Figure 9 displays the 20 most indicative keywords, with “inflammatory bowel disease” attaining the highest citation burst score of 23.66. Furthermore, terms such as “permeability” and “gene expression” have exhibited notable upward trends, indicating the rapid advancement of investigations in this domain. Figure 10 portrays the temporal development of keyword clusters, effectively illustrating the evolution of research pertaining to UC treatment through the lens of intestinal barriers. The leading ten keyword clusters within the context of intestinal barriers and ulcerative colitis are enumerated as follows: “#0 new target,” “#1 reducing inflammation,” “#2 intestinal epithelial barrier dysfunction,” “#3 traditional Chinese medicine,” “#4 protective mechanism,” “#5 induced barrier dysfunction,” “#6 ingibiting state3 palmitoylation,” “#7 gut homeostasis,” “#8 kuijie decotion,” and “#9 inhibiting inflammation.” This analysis underscores the pivotal role of the intestinal barrier in ulcerative colitis treatment and highlights the forefront of associated research endeavors.

### 3.7 Analysis of journals

Table 6 enumerates the leading ten journals that have produced the highest volume of publications in the domain of gut barrier and UC research. Collectively, these journals have disseminated a total of 550 articles, which constitutes 35.39% of the overall 1,554 publications recorded. Topping the list is the “frontiers in pharmacology,” which has garnered 1,012 citations and published 78 articles pertinent to this research area. Although “Food & Function” and “Inflammatory Bowel Diseases” do not exhibit the highest citation rates, they play a crucial role within the scholarly community and serve as vital references in this field.

**Table 6:** The top 20 keywords in terms of publications.

Rank	Keywords	Count	Centrality	Rank	Keywords	Count	Centrality
1	Ulcerative colitis	1,349	0.03	11	Metabolism	448	0.04
2	Mouse model	721	0	12	Intestinal barrier	409	0
3	Inflammatory bowel disease	689	0	13	Epithelial barrier	356	0
4	Gut microbiota	635	0	14	Inflammation	354	0
5	Human tissue	579	0.1	15	Interleukin	327	0.11
6	Animal experiment	571	0.06	16	Protein expression level	319	0.1
7	Mucus barrier	497	0.15	17	TNF-α	294	0.02
8	Disease models	491	0.02	18	Drug efficacy	278	0.2
9	Controlled study	487	0.05	19	Pathology	276	0.05
10	Tight junction	480	0	20	Gene expression	275	0

**Figure 11:**
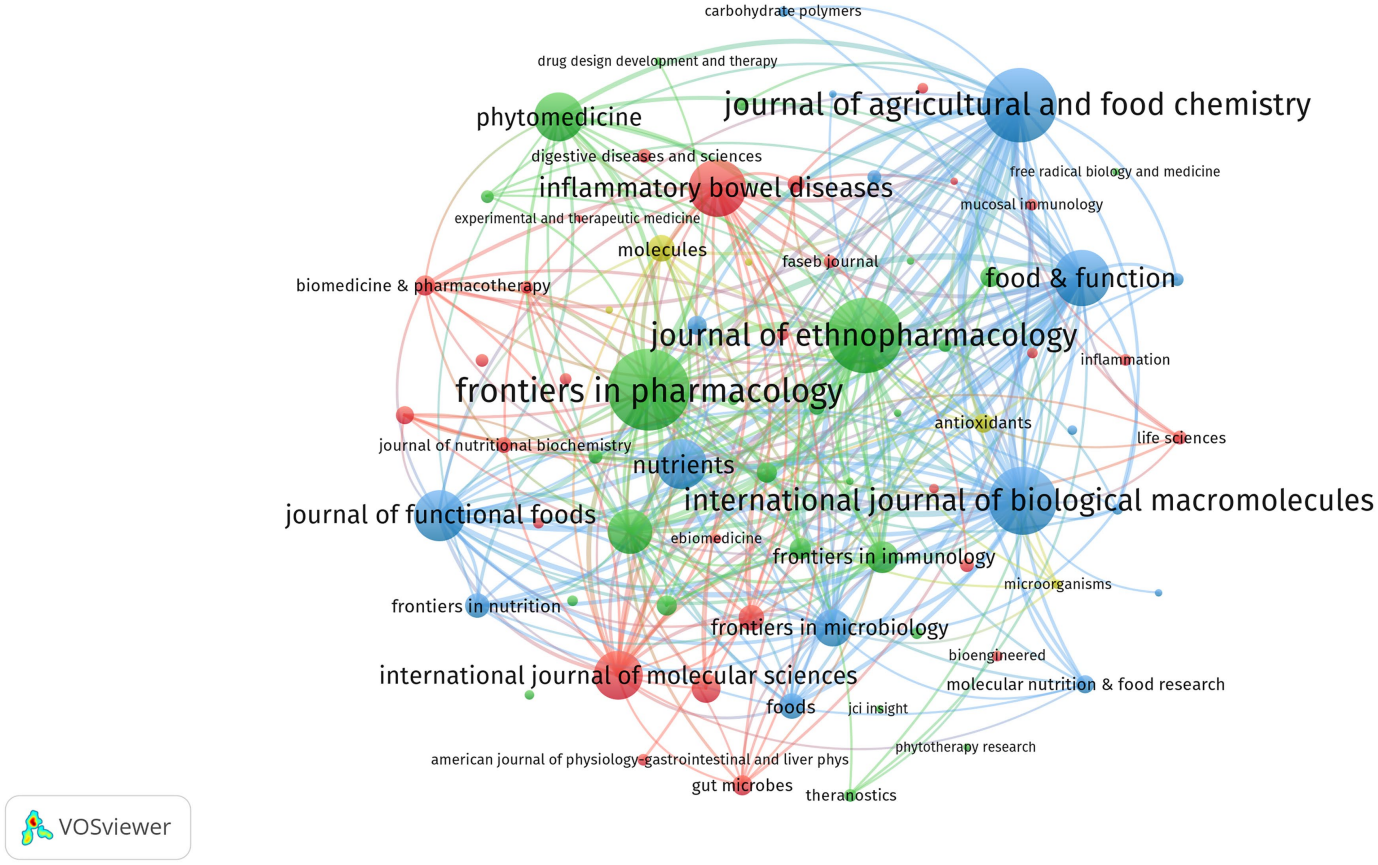
The network map of journals. The color of the nodes represents different clusters, while the size of the nodes corresponds to the number of published articles, with larger nodes indicating a greater volume of publications. The lines between nodes represent the number of co-cited references, with thicker lines signifying a higher number of co-cited references between the two journals.

**Figure 12:**
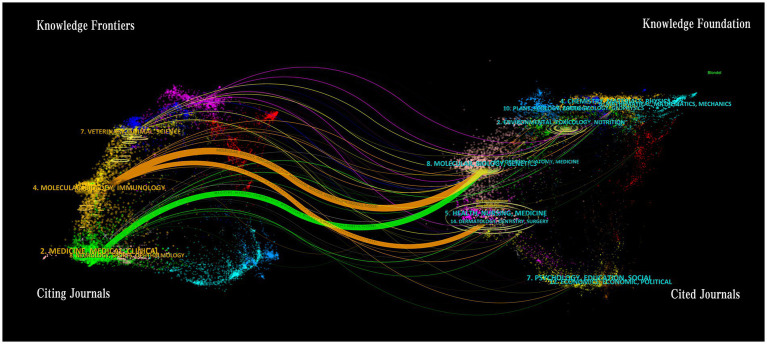
The dual-map overlay of journals. The citing journals are positioned on the left, while the cited journals are located on the right. The labels indicate the disciplines covered by the journals, and the colored paths represent the citation relationships.

Figure 11 showcases a comprehensive compilation of 1,554 articles pertaining to gut barrier and UC research, sourced from 312 journals globally, all of which have published more than 10 papers between the years 2011 and 2024. The dual-map overlay of journals depicts the co-occurrence network of disciplines, elucidating the interrelations between journals and those that are co-cited. As illustrated in Figure 12, this map delineates two predominant citation pathways that facilitate the tracing of knowledge transfer within intestinal barrier research in the context of UC studies. The left section of the figure illustrates the principal distribution groups of relevant journals as identified in the WoSCC and Scopus database. Research efforts are predominantly concentrated in the domains of cytology, immunology, molecular biology, and pharmacology. The orange pathway signifies articles produced in the realms of molecular biology and immunology, primarily cited by journals specializing in molecular biology and genetics. Conversely, the green pathway highlights research in pharmacology and clinical medicine, with findings largely referenced by journals focusing on molecular biology, genetics, health, nursing, and medicine.

## 4 Discussion

### 4.1 Fundamental information

This investigation comprehensively elucidates the evolving landscape of research pertaining to the intestinal barrier in the context of UC treatment, spanning the years 2011 to 2024, through a multifaceted bibliometric analysis. The analysis shows that the annual publication output in this field has surged dramatically after 2020. Firstly, as our understanding of UC pathogenesis deepens, we have come to recognize the crucial role of intestinal barrier dysfunction in disease progression (14). Consequently, related research has gained significant attention in the scientific community. Secondly, the outbreak of COVID-19 has made gut health, immune regulation, and chronic inflammatory diseases hot topics of research (15, 16). This has indirectly sparked interest in research on IBD, including UC. In addition, advances in technology, particularly single-cell RNA sequencing and gut microbiome analysis, have changed the way we understand the complex relationship between UC and intestinal barrier function (17, 18). Based on the above reasons, we believe that the increase in publications after 2020 is not merely a quantitative accumulation, but also a substantial expansion in both the depth and breadth of research within this domain.

International cooperation is primarily centered around China and the United States, and a robust collaborative network has been established on a global scale. The United States is renowned for its well-developed scientific research infrastructure and numerous world-class research institutions. Meanwhile, China has experienced a significant surge in investment in scientific research. This has not only provided substantial financial support for basic research but also attracted international researchers to participate. In the future, their cooperative networks are poised to evolve in increasingly diverse and multifaceted ways.

Analysis of high-frequency keywords suggests a notable transition in cutting-edge research from the exploration of fundamental mechanisms to the clinical application of findings. Early research primarily focused on mechanisms of barrier disruption, such as NF-κB signaling, oxidative stress, and TJs. More recently, the focus has shifted to exploration of clinically effective treatments, particularly the clinically oriented concepts of traditional medicine and its derivatives. For example, Natural-derived nanomedicines significantly enhance UC treatment efficacy by balancing the intestinal barrier and immune microenvironment (19–21). They are safe, with a stable and reproducible preparation process, and show potential for clinical translation. This suggests that a paradigm shift is currently taking place, moving from merely understanding disease mechanisms to developing treatment strategies focused on restoring intestinal barrier function (Table 7).

**Table 7:** Top 10 journals in terms of publications.

Ranking	Journal	Citations	Publications
1	Frontiers in Pharmacology	1,012	78
2	Journal of Ethnopharmacology	650	71
3	Food and Function	1,476	70
4	International Journal of Biological Macromolecules	665	63
5	Inflammatory Bowel Diseases	1,144	51
6	Journal of Agricultural and Food Chemistry	940	50
7	Journal of Functional Foods	429	45
8	International Journal of Molecular Sciences	383	42
9	Phytomedicine	345	42
10	International Immunopharmacology	334	38

In recent years, China has emerged as the world’s largest producer of scientific research (22). The impact of academic achievements is typically measured by citation frequency (23). However, among the top 10 most cited papers globally, only one originates from China. The main reasons for this phenomenon can be summarized as follows. First, the language barrier poses a significant challenge. Since international mainstream journals predominantly publish in English, many Chinese scholars face limitations in global visibility due to either non-standard English writing or a preference for publishing in Chinese. Secondly, there is a disparity in journal distribution. Chinese scholars predominantly publish in international regional or multidisciplinary journals rather than top publications like Nature or Science. Given that the average citation count of these platforms is relatively low, this further restricts the potential citation impact of their work. Thirdly, there exists a cognitive bias within the international academic community. Scholars from Europe and America tend to cite research within their familiar spheres, predominantly authored by native English speakers, resulting in a certain degree of neglect towards Chinese research. Moreover, a higher Impact Factor (IF) generally indicates a higher average citation frequency for a journal, but it should not be considered the sole criterion for evaluating a researcher’s academic standing. For instance, the paper by Parada Venegas D et al., entitled “Short Chain Fatty Acids (SCFAs)-Mediated Gut Epithelial and Immune Regulation and Its Relevance for Inflammatory Bowel Diseases,” was published in a journal with a modest impact factor. Nevertheless, it ranks among the most frequently cited articles in its field, underscoring its substantial academic impact and the broad international recognition of its findings. This strongly demonstrates the significant academic value of the study and the recognition it has received from the international research community. And it illustrates that the IF does not directly reflect the academic quality or practical influence of an individual paper. The advancement of theory has also led to progress in clinical practice. Highly cited literature and hot topics highlight areas where evidence has been systematically accumulated, forming the basis for clinical trials. Currently, several major biopharmaceutical companies are currently conducting clinical trials of drugs targeting the intestinal barrier (Table 8). In summary, the frontiers research is undergoing a significant shift from exploring fundamental mechanisms to the clinical application of research findings.

**Table 8:** Emerging targeting the intestinal barrier drugs for UC.

Company	Product	Phase	Indication	Trial number	Study completion
MRM Health	MH002	2b	Mild–moderate UC	2023–503,657–99-00	Completed
Microbiotica	MB310	1b	UC	NCT06582264	2026–01
Insilico Medicine	ISM5411	1a	IBD	NCT06012578	Completed
AllianThera Biopharma	ATB102	1	Moderate–severe UC	Unknown	Unknown
Immunic	IMU-856	1	Celiac Disease, UC, CD	ACTRN12620000901909	Completed

All information regarding clinical trials of drugs for maintaining the intestinal barrier in UC was obtained from www.clinicaltrials.gov, https://www.clinicaltrialsregister.eu, https://www.anzctr.org.au. UC, ulcerative colitis; CD, Crohn’s disease; IBD, inflammatory bowel disease.

The characteristics of journal distribution further validate the trend toward interdisciplinary convergence. While gastroenterology journals continue to dominate the field, nearly 35% of high-impact research is disseminated through journals in materials science, immunology, and bioengineering, underscoring the increasing significance of technology-oriented investigations.

Future investigations ought to prioritize several key areas. Primarily, there is a need to enhance the amalgamation of interdisciplinary methodologies, for instance, by advancing dynamic tracing technologies aimed at scrutinizing the spatiotemporal changes in barrier integrity. Furthermore, refining the model of international collaboration will facilitate the optimal utilization of regional resources. Lastly, it is imperative to establish experimental frameworks that more accurately reflect human pathological characteristics, thereby addressing the inadequacies of existing animal models in replicating the heterogeneity of barriers. Through innovative multidimensional approaches and collaborative efforts, it is anticipated that a seamless transition from mechanistic exploration to clinical application will be achieved, thereby paving new avenues for the precise treatment of UC.

### 4.2 Knowledge base

The intestinal barrier serves as the primary defensive mechanism of the host, playing an essential role in safeguarding against luminal pathogenic antigens and potentially harmful microorganisms. It can be categorized into four principal components based on its various functions: the physical barrier, the chemical barrier, the immune barrier, and the microbial barrier (24).

The physical barrier is structurally constituted of the mucous layer, intestinal epithelial cells (IECs), and their intercellular TJs (25). The mucus layer is regarded as the main protective barrier of the colon and has long been recognized as a vital component of the intestinal defense system. Any disruption to its structural integrity may significantly contribute to the worsening of disease progression (26, 27). Goblet cells are responsible for secreting this mucus layer, with mucin-2 (MUC2) being its primary constituent. Studies have shown a reduction in the number of goblet cells and a decline in MUC2 expression in biopsy samples from patients with UC (28). In patients experiencing active UC, the inner mucus layer has been observed to demonstrate markedly increased permeability, with certain areas exhibiting thinning or even a complete lack of mucus (29–31).

The continuous monolayer of IECs, which comprises absorptive cells, goblet cells, and microfold cells, is a vital element of the physical barrier (32, 33). As a selective barrier, it effectively separates the contents of the intestinal lumen from the underlying host connective tissue (34). The epithelial cells and their TJs obstruct the paracellular pathway, thus maintaining the integrity of the barrier (35). The structure of TJs is an adhesion complex composed of various transmembrane proteins, including Occludin, claudins, and ZO-1, which are dynamically regulated by extracellular signals. This architecture efficiently hinders the translocation of pathogens, endotoxins, and other deleterious substances from the intestinal mucosa into the bloodstream (36–38).

Research indicates that pro-inflammatory cytokines, such as tumor necrosis factor-*α* (TNF-α) and interferon-*γ* (IFN-γ), can enhance the transcription and expression of claudin-2 in IECs (39). This upregulation of pore-forming TJs results in increased permeability. Therefore, change the expression through the pore-forming pathway may represent a significant mechanism that underlies the elevation of intestinal permeability (40, 41). The intestinal chemical barrier consists of various chemical substances secreted by the gastrointestinal tract, including gastric acid, digestive enzymes, lysozyme, bile, and antimicrobial peptides (AMPs) (42).

The cohesion and interplay of these barriers are essential for the proper operation of the intestinal mucosal barrier and the preservation of intestinal homeostasis (43). The effective functionality of this intestinal barrier necessitates meticulous regulation and prompt repair following injuries to avert the development of pathological inflammation (44–46). In the context of UC management, enhancing barrier function has emerged as a pivotal therapeutic objective aimed at facilitating more profound healing (47, 48). The integrity of the barrier, along with its dynamic interactions, not only protects the physiological roles of the intestinal mucosa but also underscores its crucial involvement in the pathogenesis and treatment of UC, thereby providing innovative perspectives for clinical strategies (43).

### 4.3 Research hotspots

The progression of research focal points has identified three primary trajectories: Prospects for the application of traditional Chinese medicine, the re-establishment of tight junction integrity, and the modulation of the NF-κB/oxidative stress signaling pathway.

#### 4.3.1 Traditional Chinese medicine

**Figure 13:**
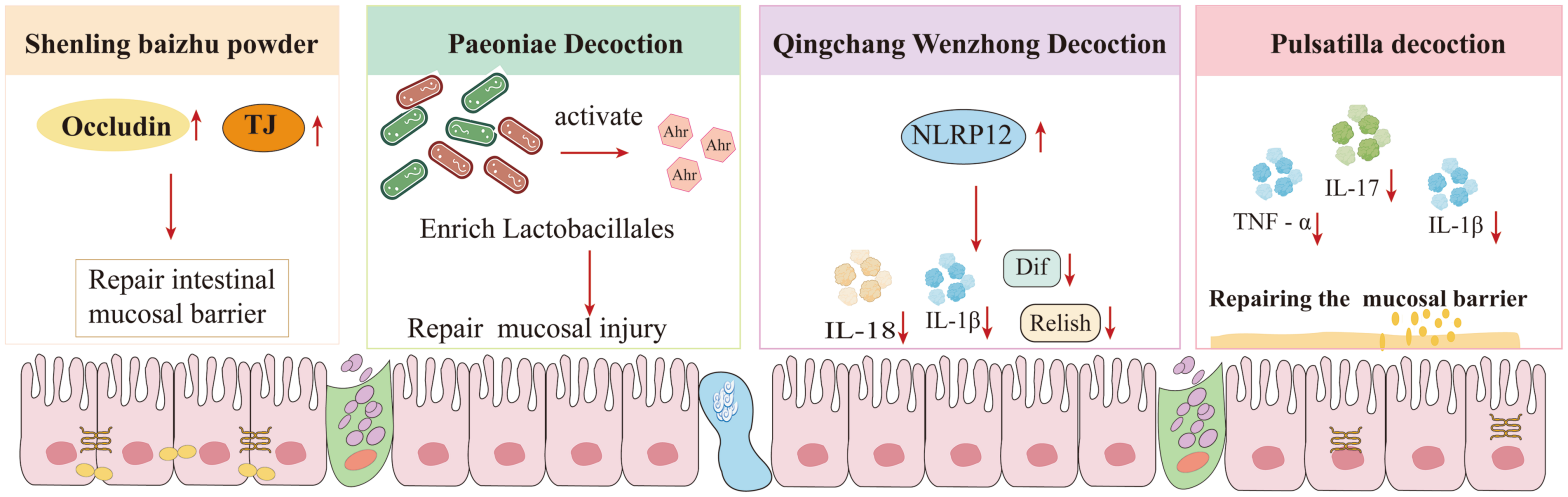
Mechanisms of action of TCM formulations on the intestinal barrier in UC.

Traditional Chinese medicine (TCM), especially formulas, have a variety of components/ingredients that exert multiple effects on UC, including intestinal mucosal repair, permeability reduction, immune regulation, and microbiome modulation (Figure 13). These comprehensive actions contribute to mucosal healing, symptom relief, disease course shortening, and recurrence prevention, ultimately achieving superior therapeutic outcomes (49). Shenling baizhu powder upregulates the levels of TJs, improves intestinal immune regulation, maintains the structural integrity of the intestinal mucosal barrier and promotes mucosal healing (50, 51). Paeoniae decoction reduces pro-inflammatory cytokine levels, modulates the gut microbiota, enhances anti-inflammatory capacity in colonic tissue, and increases the secretion of mucus to repair the mucosal epithelium (52, 53). Qingchang wenzhong decoction suppresses the TLR4/Blimp-1 axis activity regulated by gut microbiota while enhancing NLRP12 expression. This dual mechanism improves intestinal mucosal barrier function, inhibits inflammation, and effectively alleviates UC symptoms (54). Huangqin decoction upregulated the expression of Occludin and Claudin-4 while inhibiting the phosphorylation levels of p-p65 and p-IκBα, and regulated the composition of intestinal flora and amino acid metabolism. These coordinated actions maintain TJs homeostasis and ultimately alleviate intestinal epithelial barrier dysfunction (55, 56). Gegen Qinlian Decoction significantly increases colonic goblet cell numbers by promoting their proliferation and differentiation. Simultaneously, it repairs tight junction structures and enhances expression, collectively contributing to the restoration of intestinal mucosal epithelium (57–59). Pulsatilla decoction upregulates the protein expression of Occludin, ZO-1, Claudin1, Claudin5, and G protein-coupled receptor 43 (GPR43) (60).

Of course, Single-herb medicine and its extracts also demonstrate therapeutic efficacy. Research has found that the active components in *Pogostemon cablin* can significantly increase the relative abundance of probiotics, thereby regulating the balance of the microbiota and maintaining the function of the intestinal mucosal biological barrier (61). Aloe significantly enhances the expression of mucins(like MUC2 and MUC5AC). This increases the thickness of the colonic mucosa, thereby promoting the repair of the intestinal mucosa (62). Curcumin also improves intestinal barrier integrity and contributes to clinical remission in UC treatment (63–65). Tannic acid and berberine which derived from Chinese herbal medicine, can restore the expression of TJs and modulate gut microbiota composition (66). Ginsenoside Rk3 can restore colonic TJs, elevate short-chain fatty acid (SCFA) levels (acetate, butyrate, and isovalerate) and reduce pro-inflammatory cytokines (TNF-*α*, IL-1β, and IL-6) (67). Both ganoderic acid and hawthorn pectin significantly reduce intestinal inflammation and improve gut barrier function (68, 69).

Therefore, TCMs regulate intestinal function through intricate mechanisms. Although the intestinal mucosal repair mechanisms of TCMs have not been completely and systematically detailed, their reparative effects are already evident. There is also nano-sized drug deliver technology that can be used in the intestine, another avenue researchers are using to discover their mechanisms of action. We anticipate that the comprehensive regulatory mechanisms of TCMs in maintaining intestinal health will be progressively revealed in the future.

#### 4.3.2 Signaling pathways

##### 4.3.2.1 NF-κB signaling pathway

Nuclear factor κB (NF-κB) serves as a central regulator of immune responses and plays a critical role in driving aberrant intestinal inflammation of colonic origin. Activation of the NF-κB signaling pathway leads to increased expression of pro-inflammatory cytokines, including IL-6, IL-8, IL-16, and TNF-*α*, which contribute to inflammation. Simultaneously, it upregulates genes involved in cell survival and proliferation, resulting in epithelial cell apoptosis, disruption of barrier integrity, increased intestinal permeability, and the progression of UC (70, 71). Kaempferol was found to exert immunomodulatory effects in UC mice by modulating intestinal flora and a variety of metabolites, thereby inhibiting TLR4—NF-κB signalling (72). Galactooligosaccharides (GOS) administration significantly inhibited NF-κB signaling pathways and decreased inflammatory mediator expression, leading to improved intestinal barrier function (73). Saffron polysaccharides were found to improve acute UC by modulating the STAT3/NF-κB signalling pathway and repairing the intestinal barrier function (74). Consequently, investigating the NF-κB-intestinal barrier axis and inhibiting aberrant NF-κB activation in UC could lead to more effective clinical treatments.

##### 4.3.2.2 Oxidative stress signaling pathway

Oxidative stress arises when there is an imbalance between the generation of oxidants and the antioxidant defense mechanisms, which can lead to detrimental effects on biological systems (75). Central to the oxidative stress signaling pathway are reactive oxygen species (ROS) and reactive nitrogen species (RNS). In the context of mucosal inflammation, an overabundance of ROS can trigger various detrimental processes, including protein denaturation, lipid peroxidation, DNA damage, apoptosis, ferroptosis, and pyroptosis within intestinal tissues. Such alterations compromise TJs, subsequently increasing intestinal permeability (76). ROS induce profound cellular and molecular damage, perpetuate chronic intestinal inflammation, and ultimately compromise epithelial barrier integrity in UC (77, 78). Research indicates that the inhibition of macrophage polarization can diminish ROS production, lower pro-inflammatory cytokine levels, and mitigate intestinal inflammation (79, 80). Oral antioxidants have been found to reduce excess ROS in the gut and improve gut barrier function, thereby inhibiting inflammatory responses in the gut and systemically (81). In summary, oxidative stress represents a pivotal pathogenic mechanism driving both the initiation and progression of UC. Targeting this pathway through antioxidant-based therapeutic strategies may offer novel treatment paradigms for UC management.

### 4.4 Strengths and limitations

This study helps the researcher to understand the field better and explore new research directions. However, there are some limitations. First, because of software limitations, we focused solely on publications English publications, excluding nonEnglish publications, which may introduce publication bias. Although data from the WoSCC and Scopus databases were employed, the differences between these datasets could not be entirely eliminated. This limitation may potentially result in some imperfections within the integrated data. Second, citation data are subject to temporal influence, with older papers frequently receiving more citations than newer papers. Therefore, some outstanding papers might not receive their due recognition because of their relatively shorter availability. Furthermore, the common practice of citing review articles rather than original research may introduce bias. Moreover, using different combinations of literature analysis software may lead to omission of information, causing slight variations in the results. Despite these limitations, this study offers valuable insights into the evolution, hotspots, and emerging areas of intestinal barrier-based UC therapies. These insights thereby facilitate further research.

## 5 Conclusion

This study highlights the evolutionary trajectory of intestinal barrier research, which has evolved into a highly interdisciplinary field focusing on pivotal mechanisms including microbial homeostasis, tight junction reconstitution, and inflammatory cascade regulation. To bridge the gap between bench discoveries and bedside applications, we emphasize the urgent need for cross-disciplinary collaboration to translate mechanistic discoveries into clinical applications. Such integrative approaches will not only optimize therapeutic strategies but also accelerate the development of precision medicine paradigms for UC management through synergistic innovation across basic, translational, and clinical research domains.

## Data Availability

The datasets presented in this study can be found in online repositories. The names of the repository/repositories and accession number(s) can be found in the article/supplementary material.
